# A Comparison of the Asleep-Awake Technique and Monitored Anesthesia Care During Awake Craniotomy: A 10-Year Analysis

**DOI:** 10.7759/cureus.50366

**Published:** 2023-12-11

**Authors:** Ignacio Ladrero Paños, David Rivero Celada, Paula Jarén Cubillo, Cristina Bueno Fernández, Pedro Osorio Caicedo, Roberto Gomez Gomez

**Affiliations:** 1 Department of Anesthesiology, Critical Care and Pain Medicine, Miguel Servet University Hospital, Zaragoza, ESP; 2 Department of Neurosurgery, Miguel Servet University Hospital, Zaragoza, ESP; 3 Department of Anesthesiology, Critical Care and Pain Medicine, Guadalajara University Hospital, Guadalajara, ESP; 4 Department of Neurophysiology, Miguel Servet University Hospital, Zaragoza, ESP

**Keywords:** adult brain tumor, scalp block, neuro anesthesia, awake surgery, retrospective observational study

## Abstract

Background

Awake intracranial surgery with direct electrical stimulation (DES) is considered the gold standard for the resection of tumors affecting the eloquent areas of the brain. Awake craniotomy is a challenge for the anesthesiologist, as the patient's active cooperation is required throughout the operation. There are two frequent techniques, one is asleep-awake-asleep (AAA), and the other is called monitored anesthesia care (MAC). The AAA technique is the longer standing of the two and comprises general anesthesia followed by intraoperative awakening, which is necessary for neurological monitoring. In the present study, a comparison was made between the asleep-awake (AA) technique, a variation of the AAA anesthesia technique, and the MAC, which consists of a sedation that makes it possible to control pain and anxiety. Unlike the AA technique, the MAC does not involve the use of invasive airway devices.

Objective

The main objective was to contrast the two anesthetic management techniques for awake brain surgery used in our hospital.

Methods

A retrospective observational single-center study was performed consisting of a review of patient clinical records. The study sample comprised all patients above 18 years of age undergoing brain surgery through awake craniotomy between January 2013 and December 2022 at the Miguel Servet University Hospital (HUMS) in Zaragoza (Spain).

Results

Of the 79 patients included in the study, 39 were operated under AA anesthesia while the remaining 40 were operated under the MAC procedure. The main age of the participants was 52.8 years, the mean height was 169 cm, and the mean weight was 74.2 kg. No statistically significant differences were observed with respect to the patients’ baseline characteristics, except for obesity which was more prevalent in the MAC group. In the MAC group, the airway was managed by means of nasal cannulas in all cases, with conversion to general anesthesia being required in only one instance. In the AA group, the laryngeal mask (LM) was used in 89.7% of the patients, and the endotracheal tube (ETT) in 10.3%. The surgical and anesthetic procedure duration was 15 and 20 minutes shorter in the MAC group, respectively. A reduction of almost 20 minutes in the anesthetic procedure and 15 minutes in the surgical one was observed. Tachycardia, desaturation, and airway complications were observed in four, five, and four patients respectively in the AA group but in none of the patients in the MAC group. The mean stay in the intensive care unit (ICU) and the mean postoperative hemoglobin levels between both groups were insignificant.

Conclusions

Both techniques analyzed in this study turned out to be equally safe and effective for brain tumor surgery in awake patients.

## Introduction

Awake brain surgery with direct electrical stimulation (DES) is considered the gold standard for the resection of tumors located at, or close to, the eloquent areas of the brain [1]. As compared with craniotomy under general anesthesia, awake craniotomy provides for more extensive tumor resection, minimizing the risk of postoperative neurological impairment [2]. Awake craniotomy is indicated for completion of the intraoperative mapping and the subsequent resection of tumors in the eloquent area (mainly the language-related areas and the primary sensorimotor cortex) [3]. The technique is also indicated for epilepsy surgery, deep brain stimulation, and cerebrovascular procedures such as brain aneurysm embolization and arteriovenous malformation obliteration [4]. The surgical approach and the positioning of the patient depend on the location of the tumor [5]. The DES is performed to identify the sensory and motor cortices and the corticospinal tracts, as well as to locate the areas of the brain involved in the various aspects of language [6].

Awake craniotomy remains a significant challenge for anesthesiologists as active and prolonged cooperation with the patient is needed during the surgical procedure. Keeping the patient awake and monitoring their vital signs throughout the procedure comes at a price, as patients are subject to a variety of intraoperative stress factors [7]. The combination of analgesic and sedative intravenous (IV) drugs with local anesthesia techniques is highly advantageous for this kind of surgery. The most employed anesthetic techniques are asleep-awake-asleep (AAA) and monitored anesthesia care (MAC). The AAA technique consists of three anesthetic phases. In the first part of the surgery, which involves incision of the skin and the creation of a window in the cranial bone, the patient is kept under general anesthesia. During this phase, the airway is controlled with a laryngeal mask (LM) or endotracheal tube (ETT). In the second part of the procedure, before opening the dura mater, the patient must be awake to be able to cooperate and answer questions asked by the neuropsychologist during the DES. In the third and final phase, general anesthesia is used again to close the incision in the skull and skin. This is because the patient's cooperation is not required. In this study, the asleep-awake (AA) technique was used instead of AAA. The AA technique differs from AAA only in that the closure of the incision in the bone and skin is performed under sedation rather than under general anesthesia. The MAC technique consists of performing sedation that allows control of the patient's pain and anxiety without devices in the airway. This type of anesthesia allows the patient to execute commands and avoids the potential airway complications of general anesthesia. It also allows the neuropsychologist, neurophysiologist, and neurosurgeon to perform adequate cortical mapping to guarantee a correct brain resection. There is currently no consensus as to which technique allows better anesthetic management, with both AAA and MAC being considered equally safe and effective [8-10].

The Miguel Servet University Hospital (HUMS) in Zaragoza is one of the Spanish hospitals where awake intracranial surgeries are performed. The high volume of operated patients and the complexity of the procedure make it necessary to continuously evaluate the outcomes obtained. The purpose of this study was to present the hospital’s experience with the procedure over the last 10 years.

## Materials and methods

This study was carried out following approval by the Government of Aragon’s Research Ethics Committee (approval reference number C.I. EPA23/030).

A retrospective observational single-center study consisting of an analysis of patient clinical records was performed. The sample comprised all patients above 18 years of age who underwent awake intracranial surgery through craniotomy between January 2013 and December 2022 at HUMS. A total of 79 patients were operated and the anesthetic techniques were AA and MAC. Both techniques involved a scalp block (SB) with infiltration of local anesthetics, in accordance with the hospital’s standard practice.

The inclusion criteria were five: age over 18 years, brain surgery performed by awake craniotomy, elective or scheduled surgery, preoperative American Society of Anesthesiologists (ASA) physical status between I and IV, and date of surgery between 1 January 2013 and 31 December 2022 performed at the HUMS hospital. The exclusion criteria were age less than 18 years, emergency surgery, preoperative physical status ASA V and VI, and date of surgery not within the established dates.

The pre-anesthesia assessment was conducted by an anesthesiologist specializing in neuroanesthesia, with specific expertise in awake intracranial surgery. The same anesthesiologist was in charge of administering the anesthesia in the operating room. Patients were also examined by a neuropsychologist before surgery to confirm their eligibility for awake craniotomy. All patients were operated by the same surgical team led by the same neurosurgeon, who was present at every procedure. The team also comprised a neurophysiologist specializing in awake craniotomy.

Patients were intraoperatively monitored by electrocardiogram (ECG), pulse oximetry, invasive and non-invasive blood pressure (BP) measurements, and depth of sedation analysis (using the bispectral index and capnography). All patients were implanted with a large-caliber peripheral venous catheter (16-18 G), an arterial catheter in the radial artery to monitor BP, and a central venous catheter. Patients were administered 1-2 mg of IV midazolam, 0.5-1 g/kg of IV mannitol IV, and 0.1 mg/kg of IV dexamethasone before the procedure, as required. According to the anesthesiologist’s instructions, the following anesthetics were administered by continuous perfusion, either in isolation or combined: dexmedetomidine (0.2-1 mcg/kg/h), remifentanil (0.03-0.2 mcg/kg/min) and propofol (2-4 mg/kg/h). In every case, the dose was adjusted to the desired depth of sedation (general anesthesia or conscious sedation) and to the different stages of the procedure, based on the bispectral index value and the result of the neurological examination.

Patients in the AA group received general anesthesia at the beginning of the procedure, during the craniotomy phase, and from tissue dissection to the tumor resection. In these cases, the airway was managed with an ETT or a LM. Subsequently, patients were awakened and administered conscious sedation to permit assessment of their neurological function during resection. On the other hand, patients in the MAC group received conscious sedation with the above-mentioned drugs from the outset. During conscious sedation, both groups received oxygen through nasal cannulas, and all patients were monitored by capnography. Regardless of the anesthetic technique employed, all patients were given an SB with an admixture of 30-40 ml of local anesthetics (10 ml of levobupivacaine 0.5%, 15 ml of bupivacaine 0.25% with 1:200.000 adrenaline and 10 ml of mepivacaine 1.5%), the total dose of the local anesthetic being adjusted to the patient’s weight to avoid toxicity.

After the procedure, patients were transferred to the intensive care unit (ICU) where ECG, pulse oximetry, invasive BP monitoring, and oxygen therapy through nasal cannulas were continued. They all remained in the ICU for 24-48 h and were subsequently discharged to the neurosurgery department and, eventually, to their own home.

Data was collected from the clinical records of those patients who met the inclusion criteria and subsequently recorded in a spreadsheet specifically designed for the study. The information gathered included basic patient data, clinical data, analytical data, anesthesia data, and data related to the surgical procedure, complications, etc., all of which were subsequently evaluated and analyzed. The clinical records used as sources of information throughout the study period were both in paper and in digital format, with special emphasis being placed on the surgical protocol, the anesthesia records, and the analytical and biochemical parameters collected perioperatively.

The statistical analysis was carried out using Statistical Package for the Social Sciences (IBM SPSS Statistics for Windows, IBM Corp., Version 25.0, Armonk, NY). Qualitative variables were described by means of the distribution of frequencies and percentages in each category. Quantitative variables were expressed as measures of central tendency or dispersion, depending on whether they followed a normal distribution or not, as determined by the Kolmogorov-Smirnov test. Graphic representations were made using bar and pie graphs.

The association between two qualitative variables was determined with the help of contingency tables, with Pearson’s chi-squared test and Fisher's exact test being applied when necessary. Comparisons of dichotomous qualitative and quantitative variables were made by means of Student’s t-test for independent samples. The effect was determined as the difference of means and the 95% confidence interval was calculated.

Statistical significance was set at a p-value < 0.05.

## Results

Of the 79 patients included in this study, 39 were operated under the AA technique, and the remaining 40 under the MAC protocol. Table 1 shows the distribution of age, height, weight, body mass index (BMI), sex, ASA score, and preoperative baseline characteristics.

**Table 1:** Patients’ demographic variables and preoperative baseline characteristics ST: standard deviation; p^a^: Student’s t-test (comparison of means); p^b^: chi-squared test (comparison of proportions); *: statistical significance; BMI: body mass index; ASA: American Society of Anesthesiologists; HBP: high blood pressure

Demographic variables	Asleep-awake	Monitored anesthesia care	p
Age [mean (ST)]	51.2 (12.5)	54.4 (12)	0.636^a^
Height [mean (ST)]	1.7 (0.07)	1.7 (0.07)	0.541^a^
Weight [mean (ST)]	72.6 (11.1)	75.8 (18.4)	0.898^a^
BMI [mean (ST)]	24.9 (3.81)	25.9 (4.67)	0.910^a^
Sex male [n (%)]	22 (45.8)	26 (54.2)	0.434^b^
Sex female [n (%)]	17 (54.8)	14 (45.2)	0.434^b^
ASA score I [n (%)]	2 (2.5)	5 (6.3)	0.392^b^
ASA score II [n (%)]	32 (40.5)	28 (35.4)	0.392^b^
ASA score III [n (%)]	5 (6.3)	7 (8.9)	0.392^b^
Obesity [n (%)]	4 (5.3)	13 (17.3)	*0.022^b^
No obesity [n (%)]	32 (42.7)	26 (34.7)	*0.022^b^
Smoking [n (%)]	6 (7.8)	10 (13)	0.343^b^
No smoking [n (%)]	31 (40.3)	30 (39)	0.343^b^
HBP [n (%)]	9 (11.4)	10 (12.7)	0.842^b^
No HBP [n (%)]	30 (38)	30 (38)	0.842^b^
Previous respiratory disease [n (%)]	5 (6.4)	4 (5.1)	0.663^b^
No previous respiratory disease [n (%)]	33 (42.3)	36 (46.2)	0.663^b^

The mean age in the AA group was 51 years, the mean height was 169 centimeters (cm), the mean weight was 72.6 kilograms (kg), and the mean BMI was 25 points. A total of 56.4% of patients operated under this anesthesia technique were male, and 43.6% were female. In the MAC group, the mean age was 54 years, the mean height was 170 cm, the mean weight was 75.8 kg, and the mean BMI was 26 points. Sixty-five percent of the 40 patients in this group were male and 35% female. Most patients in both groups had an ASA score of II. Of all the patients in the AA group, six smoked, nine suffered from high blood pressure (HBP), and five presented with some kind of respiratory condition. Ten of the 40 patients in the MAC group smoked, 10 suffered from HBP, and four presented with respiratory conditions.

Thirty-nine percent of patients operated with the AA anesthesia technique had a history of cognitive impairment, 49% had suffered an epileptic seizure and 74% were being treated with antiepileptic drugs (AEDs) before undergoing surgery. In the MAC group, 35% of patients had a history of preoperative cognitive impairment, 50% had suffered epileptic seizures and 60% were on some kind of AED.

No statistically significant differences were found among the patients included in the study with respect to their preoperative baseline characteristics, except for obesity, which was defined as BMI ≥ 30. Nor were any statistically significant differences observed with respect to preoperative neurological impairment or the characteristics of the operated brain tumors.

During the intraoperative period, LM was used in 89.7% of patients in the AA group to manage the airway, whereas ETT was used in 10.3% of cases. Nasal cannulas were used in 100% of patients in the MAC group. Over half of the patient sample was operated in the supine position and language function was the most commonly used indicator to monitor the patient’s neurological status in both groups, followed by motor function. Statistically significant differences were observed between the airway management devices used during the procedure. A nearly statistically significantly higher conversion rate to general anesthesia was observed in the AA group (Table 2). No differences were found between the groups with respect to patient positioning or neurological monitoring.

**Table 2:** Airway, conversion, patient positioning, and monitoring during the procedure p^a^: chi-squared test (comparison of proportions); *: statistical significance; LM: laryngeal mask; ETT: endotracheal tube

Variables	Asleep-awake [n (%)]	Monitored anesthesia care [n (%)]	p^a^
LM	35 (89.7)	0 (0)	< 0.001*
ETT	4 (10.3)	0 (0)	< 0.001*
Nasal cannula	0 (0)	40 (100)	< 0.001*
Conversion to general anesthesia	5 (6.3)	1 (1.3)	0.083
No conversion to general anesthesia	34 (43)	39 (49.4)	0.083
Lateral patient placement	18 (48.6)	15 (37.5)	0.323
Supine patient placement	9 (51.4)	25 (62.5)	0.323
Language function monitoring	28 (73.7)	29 (72.5)	0.984
Motor function monitoring	7 (18.4)	8 (20)	0.984
Language and motor function monitoring	3 (7.9)	3 (7.5)	0.984

**Figure 1:**
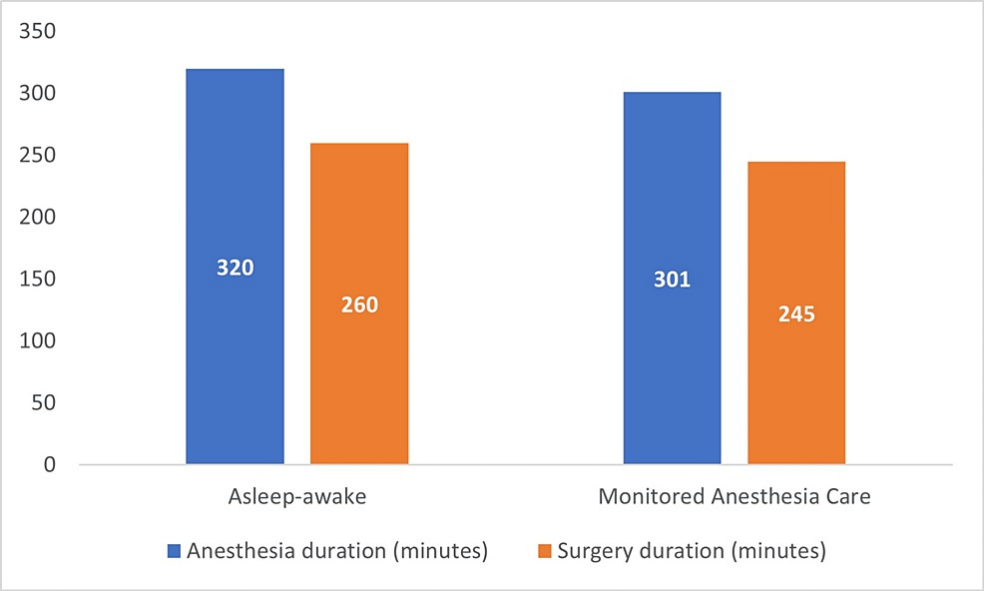
Mean duration of anesthesia and surgery

The mean duration of anesthesia and the surgical procedure was 320 and 260 minutes respectively in the AA group. In the MAC group, the mean anesthesia time was 301 minutes, and the mean surgical time was 245 minutes. No statistically significant differences were found (Figure 1).

Dexamethasone was used in 87.2% of patients in the AA group and mannitol in 82.1%. The mean dose of midazolam administered was 1.33 mg. In the MAC group, dexamethasone was used in 60% of patients and mannitol in 82.1%. The mean dose of midazolam administered was 1.36 mg.

Table 3 shows that 35.9% of patients in the AA group presented at least one episode of hypotension, while 15.4% experienced HBP, 2.6% bradycardia, and 10.3% tachycardia at some point.

**Table 3:** Hemodynamic alterations and complications during the procedure p^a^: chi-squared test (comparison of proportions); *: statistical significance; IONV: intraoperative nausea and/or vomiting; HBP: high blood pressure

Hemodynamic alterations	Asleep-awake [n (%)]	Monitored anesthesia care [n (%)]	p^a^
Hypotension	14 (35.9)	10 (25)	0.292
No hypotension	25 (64.1)	30 (75)	0.292
HBP	6 (15.4)	5 (12.5)	0.711
No HBP	33 (84.6)	35 (87.5)	0.711
Bradycardia	1 (2.6)	3 (7.5)	0.317
No bradycardia	38 (97.4)	37 (92.5)	0.317
Tachycardia	4 (10.3)	0 (0)	0.038*
No tachycardia	35 (89.7)	40 (100)	0.038*
Desaturation	5 (12.8)	0 (0)	0.019*
No desaturation	34 (87.2)	40 (100)	0.019*
Airway complications	4 (10.5)	0 (0)	0.035*
No airway complications	34 (89.5)	40 (100)	0.035*
Pain during the awake stage	2 (5.1)	5 (12.5)	0.249
No pain during the awake stage	37 (94.9)	35 (87.5)	0.249
IONV	1 (2.6)	3 (7.5)	0.317
No IONV	38 (97.4)	37 (92.5)	0.317
Restlessness	1 (2.6)	1 (2.5)	0.986
No restlessness	38 (97.4)	39 (97.5)	0.986

Twenty-five percent of patients in the MAC group presented with hypotension at some point during surgery, 12.5% HBP, and 7.5% bradycardia. No patient exhibited intraoperative tachycardia, with statistically significant differences between the groups.

During the intraoperative period, 12.8% of patients in the AA group experienced an episode of desaturation and 10.5% exhibited airway complications. None of the patients in the MAC group exhibited either of these two events, with statistically significant differences being found between both variables.

Two episodes of verbally reported pain were recorded intraoperatively in the AA group. One patient presented with intraoperative nausea and/or vomiting (IONV) and another exhibited restlessness. In the MAC group, five patients experienced pain at some point during the intraoperative period, three experienced IONV, and one suffered an episode of restlessness.

**Figure 2:**
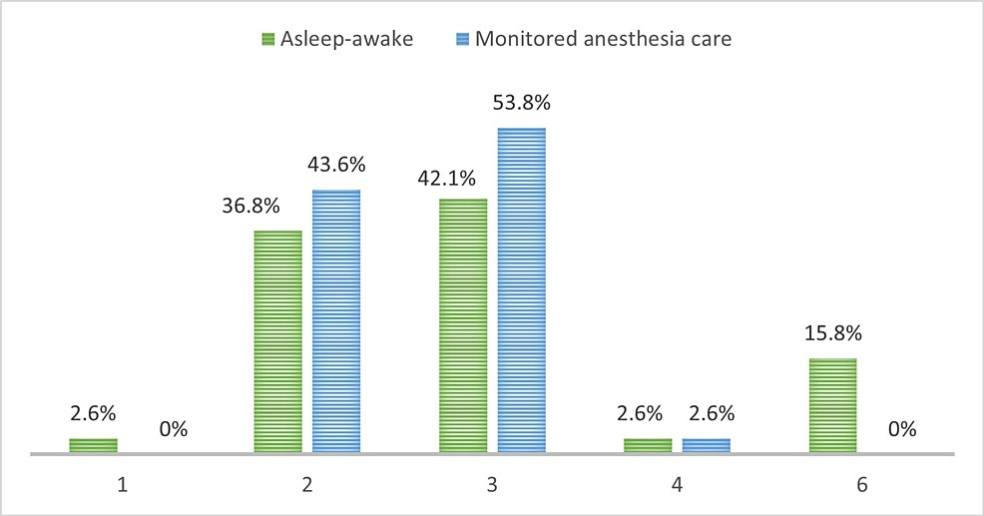
Levels of sedation on admission to the ICU, as measured by the Ramsay scale

During the intraoperative period, 36.8% of patients in the AA group had a sedation level of 2 on the Ramsay scale on admission to the ICU, 42.1% had a sedation level of 3, and 15.8% had a level of sedation of 6. In the MAC group, 43.6% of patients had a sedation level of 2, 53.8% had a level of 3 and no patient had a level of 6. No statistically significant differences were found. The p-value was 0.093 (Figure 2).

**Figure 3:**
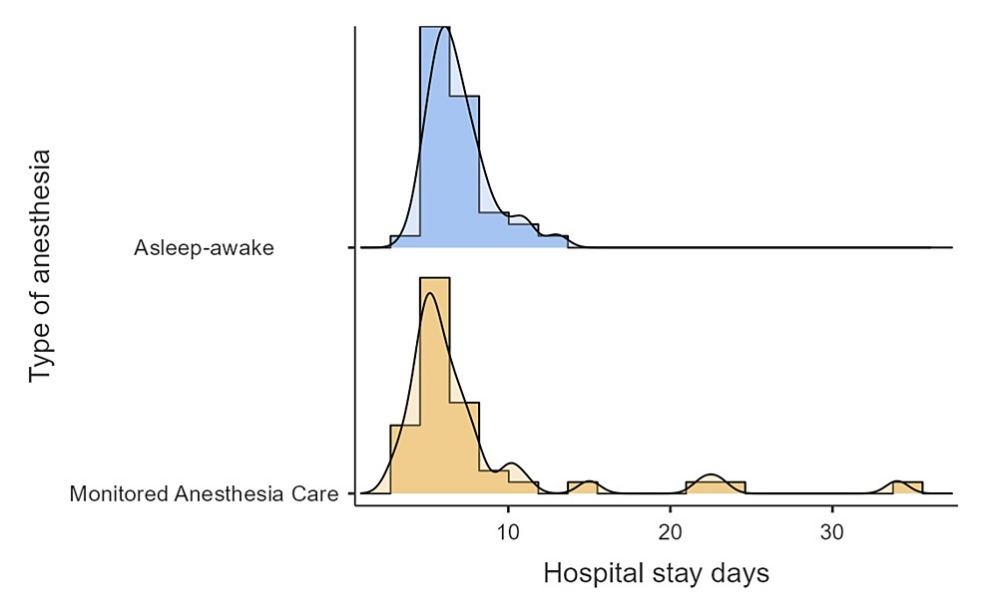
Length of hospital stay

The mean stay in the ICU for patients in the AA group was 1.05 days, with 5.1% remaining in the ICU for over 24 hours. The mean hospital stay was 6.92 days. Patients in the MAC group remained in the ICU for a mean of 1.13 days, with 10% staying in the ICU for longer than 24 hours. The mean hospital stay in this group was 7.7 days (Figure 3).

Mean postoperative hemoglobin levels on admission to the ICU in the AA group were 12.6 g/dL, with the difference between mean pre- and post-operative hemoglobin values standing at 1.98 g/dL. In the MAC group, mean postoperative hemoglobin levels were 12.6 g/dL and the difference between mean pre- and postoperative hemoglobin levels was 2.07 g/dL. No statistically significant differences were found between hemoglobin values and the length of ICU or hospital stay.

A total of 25.6% of patients in the AA group experienced some sort of (transient or long-term) worsening of their neurological status after the procedure. Thirty-five percent of patients in the MAC group presented with some kind of worsening of their neurological status. As regards the type of tumor resection performed, 61.5% of patients in the AA group were subjected to a complete resection, whereas 38.5% underwent a partial resection. In the MAC group, a complete resection was performed in 65% of patients, with the remaining 35% undergoing a partial resection (Table 4).

**Table 4:** Type of resection and postoperative neurological impairment p^a^: chi-squared test (comparison of proportions)

Resection and neurological impairment	Asleep-awake [n (%)]	Monitored anesthesia care [n (%)]	p^a^
Complete tumor resection	24 (61.5)	26 (65)	0.750
Partial tumor resection	15 (38.5)	14 (35)	0.750
Postoperative neurological status worsening	10 (25.6)	14 (35)	0.366
No postoperative neurological status worsening	29 (74.4)	26 (65)	0.366

**Figure 4:**
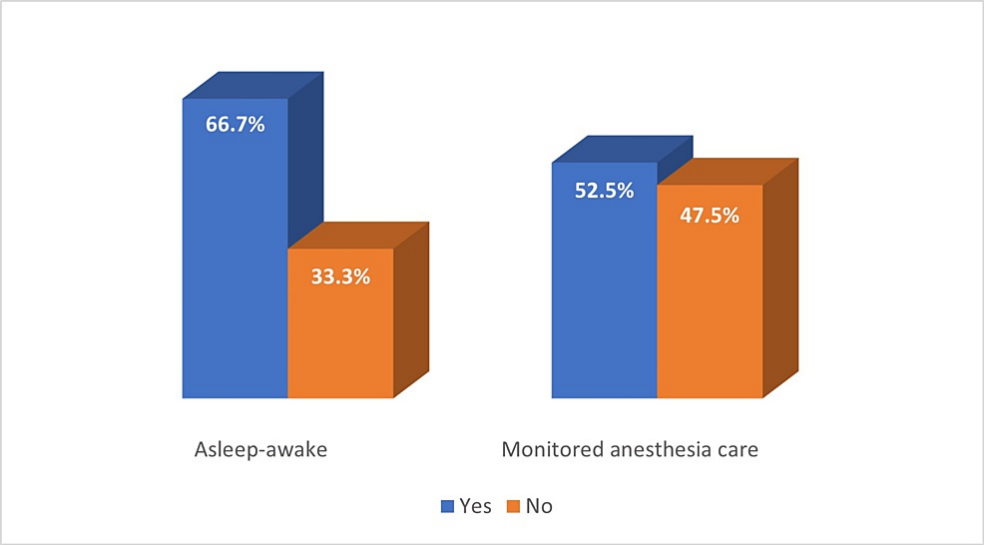
Deaths during the follow-up period

No patient in the AA group died during their stay in hospital, with a total of 66.7% passing away during the study period (by December 2022). The mean survival in this group was 20.3 months. In the MAC group, the mean postoperative survival was 14.4 months. A total of 52.5% of patients died during the follow-up period and none of them passed away during their stay in hospital (Figure 4).

## Discussion

The number of patients in each group was very similar to our sample (39 in the AA group and 40 in the MAC group). The mean patient age was around 50 years [11] and, in line with Hervey & Sacko, the majority of our subjects were ASA grade II [12]. Although the mean weight among patients in the MAC group was 3.2 kg higher than in the AA group, our results are similar to Peruzzi et al.’s in that no statistically significant differences were found with respect to weight or sex [13]. The most common comorbidities among our patients were HPB, preoperative cognitive impairment, and a history of epileptic seizures. Nearly one-third of the patients operated on using the MAC technique presented with a BMI > 30, as compared with 11% in Eseonu et al. who, unlike us, did not find statistically significant differences in this regard [14]. Conte et al. found that over half of their patients undergoing surgery had a history of epileptic seizures and were on AEDs. This coincides with the findings from the present study where a prevalence of nearly 50% was observed of previous epileptic seizures in both groups. Moreover, over 60% of patients in both groups were on some kind of AED [15].

Certain authors have argued that, in the context of general anesthesia, LM is the best option for managing the patients’ airways as it prevents the risk of oversedation and HBP. Our data bear out that hypothesis, and the most commonly used airway management device in the AA group was the LM (89% of cases) [16]. A total of 6.3% of cases in the AA group had to be converted to general anesthesia, as compared with 1.3% for the MAC group. The conversion rate among patients in the MAC group was similar to that reported by Stevanovic et al. in their metanalysis [9]. Although neither study found statistically significant differences, ours did show a trend toward statistical significance in this regard.

Careful patient positioning is a crucial factor for the success of awake craniotomy. It has been found that patient comfort during the procedure favors therapeutic compliance. Participation of patients in the positioning phase appears to result in higher satisfaction levels [17]. The most commonly used patient positions during the procedure in our study were the lateral and the supine positions. Special attention was given to patient placement as long-term uncomfortable patient positioning has been shown to be a common complaint among patients undergoing awake craniotomy [18].

As elsewhere in the literature, patients in this study were implanted with a large-caliber peripheral vascular cannula and were subjected to standard monitoring, consisting of pulse oximetry, ECG, and non-invasive BP monitoring. The arterial cannula was used to measure BP and monitor gas exchange using an arterial blood gas test. A capnography device was also used to measure expired carbon dioxide levels, and the bispectral index to determine depth of sedation. Implantation of central venous access allows rapid and safe administration of drugs for treating certain complications (seizures, cerebral edema, blood loss, and cardiopulmonary disturbances). The frequency of intraoperative neurological monitoring was similar to that described by other authors. Language monitoring was performed in around 70% of patients, while motor monitoring was carried out in 20-30% [19].

Unlike Gupta et al., we found that the mean duration of anesthesia and the surgical procedure were shorter in the MAC group, although the difference did not reach statistical significance [20]. Shorter procedures are associated with shorter hospital stays and, potentially, with a lower risk of infection and a faster wound healing rate. In spite of obtaining similar results to ours, Brown et al. could not demonstrate that shorter surgical times resulted in significant improvements in either of their two groups [21].

The selection of anesthetic agents for awake craniotomy depends on the results of the DES and the intraoperative electrocorticography. The most common drugs for conscious sedation are propofol, midazolam, remifentanil, fentanyl, and dexmedetomidine [22]. Similarly to Shen et al., our patients were sedated with dexmedetomidine, propofol, and remifentanil because of the effectiveness and favorable safety profile of these drugs. Some authors have suggested that dexmedetomidine could be the most appropriate sedative for la awake craniotomy as it is associated with a shorter awakening time and a higher degree of surgeon satisfaction [23].

The most common hemodynamic complications reported in the literature include intraoperative episodes of HBP, hypotension, and tachycardia. Like Eseonu et al., our study found no differences regarding the extent of the tumor resection; the presence of complications such as HBP or seizures; or conversion to general anesthesia with ETT [10]. Nonetheless, in line with Dilmen et al. [24], statistically significant differences were found regarding the increase in patients’ heart rate.

Despite the performance of an SB, patients may experience pain during the procedure if opioids are not administered. Up to 30% of patients reported considerable intraoperative pain. The SB may fail to appropriately cover the area of the incision, which may result in patients experiencing pain in adjacent body parts. Similarly, the block may wear off in the course of a lengthy procedure depending on the type of local anesthetic used. In line with Joswig et al., most patients in this study (> 87%) did not experience pain or restlessness during the awake stage of the procedure [25]. Similarly, and in agreement with Kawata et al., our percentage of patients with pain during awake craniotomy was slightly higher than 10% (12.5%). Our IONV rate, however, was lower (7.5%) than that reported in the literature [26,27].

According to some authors, awake craniotomy is associated with shorter ICU and hospital stays as compared with craniotomy under general anesthesia. Taylor et al. reported a mean stay of less than one day in the ICU and a mean stay of less than three days in hospital [28]. Mean ICU and hospital stay in our analysis were slightly longer in the MAC group (1.13 and 7.7 days vs. 1.05 and 6.92 respectively). This difference was due to the presence of postoperative complications in three patients in the MAC group, which increased mean hospital stay. One patient developed intraoperative aphasia, which made it necessary to suspend the procedure and carry out a second procedure; another patient suffered a postoperative ischemic stroke; and a third one developed postoperative neurological complications.

Intraoperative mapping showed itself to be highly advantageous for detecting functional areas of the brain during tumor resection. Although our study showed no differences with respect to the amount of resection determined preoperatively and the extent of the resection actually performed, Gerritsen et al. found a higher probability of complete resections in patients undergoing awake craniotomy for resection of glioblastoma in the eloquent area than in those subjected to asleep craniotomy [29]. Several authors have claimed that the incidence of permanent neurological impairment is lower among patients undergoing awake craniotomy than among those where general anesthesia is performed. This lower incidence is typically accompanied by improved quality of life and longer survival. Similarly to Suarez et al., our study found no statistically significant differences between AA and MAC with respect to neurological status worsening [30].

Our analysis did not reveal a direct relationship between survival and anesthetic management. In Gerritsen et al., awake craniotomy resulted in less neurological impairment at three months and longer overall and progression-free survival rates than craniotomy under general anesthesia [28]. Nonetheless, no individual technique has been shown to be superior to the other, with technique selection being dictated by clinical and institutional preference. Similarly to our study, a meta-analysis comparing AA with MAC found differences with respect to intraoperative seizures, worsening of neurological status, and the failure of the craniotomy [9].

Limitations

This is an observational, descriptive, single-center study, so the generalization of the data to other centers (external validity) and to the general population will be limited. As all procedures were not performed by the same anesthesiologist, differences in patient management may be found.

## Conclusions

The MAC technique was superior to the AA in the duration of intracranial surgery. It demonstrated a reduction in anesthetic and surgical time (19 and 15 minutes respectively) but no statistically significant differences were found. ICU stay was almost similar (0.08 days longer in the MAC group) and slightly longer hospital stay for the MAC group (0.78 days).

The MAC technique has proven to be a safe technique for awake craniotomy. It has shown less need for the use of advanced airway management devices and a lower incidence of airway complications and a need for conversion to general anesthesia. It has also demonstrated a lower incidence of intraoperative hemodynamic complications, such as hypo- and hypertension, tachycardia, and desaturation.

Both techniques appear to be equally safe in the context of brain tumor surgery in awake patients. However, the MAC procedure tends to be associated with fewer airway management complications and shorter surgical times.

## References

[1] McAuliffe N, Nicholson S, Rigamonti A (2018). Awake craniotomy using dexmedetomidine and scalp blocks: a retrospective cohort study. Can J Anaesth.

[2] Erickson KM, Cole DJ (2007). Anesthetic considerations for awake craniotomy for epilepsy. Anesthesiol Clin.

[3] Meng L, Berger MS, Gelb AW (2015). The potential benefits of awake craniotomy for brain tumor resection: an anesthesiologist’s perspective. J Neurosurg Anesthesiol.

[4] Szelényi A, Bello L, Duffau H (2010). Intraoperative electrical stimulation in awake craniotomy: methodological aspects of current practice. Neurosurg Focus.

[5] Tonn JC (2007). Awake craniotomy for monitoring of language function: benefits and limits. Acta Neurochir (Wien).

[6] De Witt Hamer PC, Robles SG, Zwinderman AH, Duffau H, Berger MS (2012). Impact of intraoperative stimulation brain mapping on glioma surgery outcome: a meta-analysis. J Clin Oncol.

[7] Palese A, Skrap M, Fachin M, Visioli S, Zannini L (2008). The experience of patients undergoing awake craniotomy: in the patients' own words. A qualitative study. Cancer Nurs.

[8] Kulikov A, Lubnin A (2018). Anesthesia for awake craniotomy. Curr Opin Anaesthesiol.

[9] Stevanovic A, Rossaint R, Veldeman M, Bilotta F, Coburn M (2016). Anaesthesia management for awake craniotomy: systematic review and meta-analysis. PLoS One.

[10] Eseonu CI, ReFaey K, Garcia O, John A, Quiñones-Hinojosa A, Tripathi P (2017). Awake craniotomy anesthesia: a comparison of the monitored anesthesia care and asleep-awake-asleep techniques. World Neurosurg.

[11] Keifer JC, Dentchev D, Little K, Warner DS, Friedman AH, Borel CO (2005). A retrospective analysis of a remifentanil/propofol general anesthetic for craniotomy before awake functional brain mapping. Anesth Analg.

[12] Sacko O, Lauwers-Cances V, Brauge D, Sesay M, Brenner A, Roux FE (2011). Awake craniotomy vs surgery under general anesthesia for resection of supratentorial lesions. Neurosurgery.

[13] Peruzzi P, Bergese SD, Viloria A, Puente EG, Abdel-Rasoul M, Chiocca EA (2011). A retrospective cohort-matched comparison of conscious sedation versus general anesthesia for supratentorial glioma resection. Clinical article. J Neurosurg.

[14] Eseonu CI, Rincon-Torroella J, ReFaey K, Lee YM, Nangiana J, Vivas-Buitrago T, Quiñones-Hinojosa A (2017). Awake craniotomy vs craniotomy under general anesthesia for perirolandic gliomas: evaluating perioperative complications and extent of resection. Neurosurgery.

[15] Conte V, Magni L, Songa V (2010). Analysis of propofol/remifentanil infusion protocol for tumor surgery with intraoperative brain mapping. J Neurosurg Anesthesiol.

[16] Lobo FA, Amorim P (2006). Anesthesia for craniotomy with intraoperative awakening: how to avoid respiratory depression and hypertension?. Anesth Analg.

[17] Deras P, Moulinié G, Maldonado IL, Moritz-Gasser S, Duffau H, Bertram L (2012). Intermittent general anesthesia with controlled ventilation for asleep-awake-asleep brain surgery: a prospective series of 140 gliomas in eloquent areas. Neurosurgery.

[18] Costello TG, Cormack JR (2004). Anaesthesia for awake craniotomy: a modern approach. J Clin Neurosci.

[19] Seemann M, Zech N, Graf B, Hansen E (2015). Anesthesiological management of awake craniotomy: Asleep-awake-asleep technique or without sedation [Article in German]. Anaesthesist.

[20] Gupta DK, Chandra PS, Ojha BK, Sharma BS, Mahapatra AK, Mehta VS (2007). Awake craniotomy versus surgery under general anesthesia for resection of intrinsic lesions of eloquent cortex - a prospective randomised study. Clin Neurol Neurosurg.

[21] Brown T, Shah AH, Bregy A (2013). Awake craniotomy for brain tumor resection: the rule rather than the exception?. J Neurosurg Anesthesiol.

[22] Hans P, Bonhomme V, Born JD, Maertens de Noordhoudt A, Brichant JF, Dewandre PY (2000). Target-controlled infusion of propofol and remifentanil combined with bispectral index monitoring for awake craniotomy. Anaesthesia.

[23] Shen SL, Zheng JY, Zhang J, Wang WY, Jin T, Zhu J, Zhang Q (2013). Comparison of dexmedetomidine and propofol for conscious sedation in awake craniotomy: a prospective, double-blind, randomized, and controlled clinical trial. Ann Pharmacother.

[24] Dilmen OK, Akcil EF, Oguz A, Vehid H, Tunali Y (2017). Comparison of conscious sedation and asleep-awake-asleep techniques for awake craniotomy. J Clin Neurosci.

[25] Joswig H, Bratelj D, Brunner T, Jacomet A, Hildebrandt G, Surbeck W (2016). Awake craniotomy: first-year experiences and patient perception. World Neurosurg.

[26] Kawata M, Fukui A, Mineharu Y (2022). A nationwide questionnaire survey on awake craniotomy in Japan. Neurol Med Chir (Tokyo).

[27] Oliver-Fornies P, Sánchez-Viñas A, Gomez Gomez R (2023). Cost analysis of low-volume versus standard-volume ultrasound-guided interscalene brachial plexus block in arthroscopic shoulder surgery. Cureus.

[28] Taylor MD, Bernstein M (1999). Awake craniotomy with brain mapping as the routine surgical approach to treating patients with supratentorial intraaxial tumors: a prospective trial of 200 cases. J Neurosurg.

[29] Gerritsen JKW, Zwarthoed RH, Kilgallon JL (2022). Effect of awake craniotomy in glioblastoma in eloquent areas (GLIOMAP): a propensity score-matched analysis of an international, multicentre, cohort study. Lancet Oncol.

[30] Suarez-Meade P, Marenco-Hillembrand L, Prevatt C (2020). Awake vs. asleep motor mapping for glioma resection: a systematic review and meta-analysis. Acta Neurochir (Wien).

